# Cost of chronic kidney disease attributable to diabetes from the perspective of the Brazilian Unified Health System

**DOI:** 10.1371/journal.pone.0203992

**Published:** 2018-10-01

**Authors:** Gabriela Maria Reis Goncalves, Everton Nunes da Silva

**Affiliations:** 1 University of Brasília, Brasília, Federal District, Brazil; 2 National Institute os Science and Technology for health Technology Assessment (IATS)–CNPq, Porto Alegre, Rio Grande do Sul, Brazil; Postgraduate Medical Institute, INDIA

## Abstract

**Introduction:**

Diabetes is the most common cause of chronic kidney disease, with a high economic impact on health systems.

**Objective:**

To estimate the cost of chronic kidney disease (CKD) and end-stage kidney disease (ESKD) attributable to diabetes, stratified by sex, race/skin color, and age, from the perspective of the Brazilian public health system between 2010 and 2016.

**Methods:**

Population attributable risk (PAR) was calculated from the Brazilian prevalence of diabetes and the relative risk (or odds ratio) of persons with diabetes developing CKD and ESKD as compared to non-diabetic subjects. The variables of interest were sex, race/skin color, and age. A top-down approach was used to measure the direct costs of the disease reimbursed by the Brazilian Ministry of Health, using data from outpatient and inpatient records.

**Results:**

The cost of CKD and ESKD attributable to diabetes in the period 2010–2016 was US$1.2 billion (US$180 million per year) and trending upward. Female sex, age 65–75, and black race/skin color contributed substantially to the costs of CKD and ESKD (US$475 million, US$63 million, and US$25 million respectively). The clinical procedures accounting for the greatest share of disease-attributable costs are hemodialysis and peritoneal dialysis.

**Conclusion:**

Diabetes accounted for 22% of the costs of CKD and ESKD. Female sex, age 65–75 years, and black race/skin color were the variables which contributed most to disease-related expenditure. The economic burden of CKD may increase gradually in the coming years, with serious implications for the financial sustainability of the Brazilian public health system.

## Introduction

Chronic kidney disease (CKD) is a leading public health problem worldwide due to its rising prevalence, high mortality, and high cost to health systems [1,2,3]. CKD affects 14.3% of the population in low- and middle-income countries and 13.4% of the global population [1,4]. CKD affects different population groups according to sex, age and race/color and is directly related to social and economic issues, as well as health surveillance and access to health services [5]. In Brazil, the estimated prevalence of CKD in adults is 1.4%-8.9%, similar between sexes, increasing with age and more prevalent among blacks [6,7]. The lower prevalence in Brazil compared to international data may be related to the poor quality of CKD prevalence studies in Brazil [8].

The burden of CKD is substantial. According to the World Health Organization (WHO), in 2012, 1.5% of all deaths worldwide were attributable to this condition. This means it is ranked 14th on the list of leading causes of mortality, accounting for 12 deaths per 100,000 population. Since 1990, only deaths due to complications of HIV infection have increased at a faster pace than deaths from CKD. WHO forecasts suggest that CKD mortality will continue to rise, reaching 14 deaths per 100,000 population by 2030 [2].

Chronic kidney disease (CKD) is part of the rise in noncommunicable diseases (NCDs) diabetes being the primary cause in developed and developing countries [9]. In 1980, the worldwide prevalence of diabetes was 4.3% for both sexes; by 2014, it had risen to 7.9% in women and 9% in men [10]. In Brazil, 6.2% of the population has been diagnosed with diabetes, which corresponds to 9.1 million people [6]. This marked increase in diabetes drives a rise in rates of of chronic kidney disease, leading to greater expenditure of economic resources by the health system [1].

In lower-income countries, where access to health services is scarce or nonexistent [11], screening for kidney disease in specific populations and early intervention are powerful tools that can minimize the progression of CKD and, consequently, reduce the costs associated with its treatment [9], thus allowing public resources to be redirected to other health issues.

In Brazil, 83% of CKD treatment costs are refunded by the National Health System (SUS), the Ministry of Health being the main funder [12]. Despite the high cost of CKD, few Brazilian studies have estimated federal government expenditures on kidney disease treatment [13].

Considering the scarcity of data on the economic burden of diabetes in CKD to health systems, the present study aims to estimate the cost of CKD attributable to diabetes, stratified by gender, race/skin color, and age, to the Brazilian publicly funded health system between 2010–2016. Diabetes was selected because it is the main cause of CKD, which can be potentially prevented and controlled. The findings of this study should contribute to improving the management of health system expenditures in developing countries, including Brazil.

## Materials and methods

### Study setting

This economic evaluation study was conducted using a cost-of-illness analysis design [14], from the perspective of the Brazilian Unified Health System (*Sistema Único de Saúde*, SUS), from 2010 to 2016.

The 1988 Brazilian constitution recognized health as a citizen’s right and a duty of the state, and established the basis for the creation of the Unified Health System (SUS), which was based on the principles of universality, integrality, and social participation [15]. The SUS is tasked with undertaking health promotion, health surveillance, vector control, health education, and with ensuring continuity of care to all Brazilians at the primary, specialist outpatient, and hospital levels. Also, is responsible for approximately 75% of medical care in Brazil [16]. Specifically for ESKD treatment in Brazil, 83% of the patients are cared for by the SUS and 17% by private health insurance [12].

In the SUS, secondary health care initiatives reported by public and private hospitals are denominated medium-to-high-complexity procedures, characterized by the following aspects: more advanced technology; higher costs and more specialized care. For purposes of information and funding by the Ministry of Health, the medium and high-complexity procedures are registered in the SUS Outpatient Information System (*Sistema de Informação Ambulatorial*, SIA/SUS) and Hospital Information System (*Sistema de Informação Hospitalar*, SIH/SUS).

Brazil has undergone major political, economic, demographic, and social changes in the past 40 years [15]. In 2018, according to the International Monetary Fund (IMF), Brazil had a per capita gross domestic product (GDP) of USD 10,220, classifying it as an emerging market with a developing economy. The IMF’s 2017 World Economic Outlook (WEO) report classifies countries according to economic perspective as a function of per capita GDP. Advanced economies include the USA (GDP: $62,150), Japan (GDP: $40,850) and Canada (GDP: $48,470); emerging markets and developing economies, such as Brazil, include Russia (GDP: $11,950), China (GDP: $10,090) and Mexico (GDP: $9,720); and the low-income developing countries such as Laos (GDP: $2,710), Nigeria (GDP: $2,110) and Haiti (GDP: $847.09) [17].

According to the Brazilian Institute of Geography and Statistics (IBGE), the average monthly salary for the main household wage earner in 2016 was USD 683.00, and the average monthly cost of hemodialysis is USD 660.00, not including other related costs (drugs, doctor’s appointments and transportation) [18]. For the Brazilian family, the impact of CKD is catastrophic and dependence on SUS is essential to surviving.

### Study design

Chronic kidney disease (CKD) is currently classified according to the patient’s glomerular filtration rate (GFR) and urinary albumin excretion, as the association of these two parameters with disease progression, adverse renal outcomes, and mortality is widely recognized. For purposes of economic analysis, CKD was divided into two categories for this study: chronic kidney disease (CKD) and end-stage kidney disease (ESKD).

The estimated cost of CKD and ESKD associated with diabetes was obtained by calculating the population attributable risk (PAR) by sex, race/skin color, and age, and multiplied by the costs of medium- and high-complexity procedures recorded in the SUS Outpatient Information System (*Sistema de Informação Ambulatorial*, SIA/SUS) and Hospital Information System (*Sistema de Informação Hospitalar*, SIH/SUS) from January 2010 to December 2016. The population considered for analysis was restricted to adults (age >18 years). PAR was calculated by the following formula [14]:

$$PAR=\frac{P\;(RR-1)}{P\;(RR-1)+1}x100$$

Where:

P = Prevalence of diabetes by sex, race/skin color, and age, as obtained from the National Health Survey [6], and

RR = Relative risk that persons with diabetes will develop CKD and ESKD versus persons without diabetes, by sex, race/skin color, and age.

To calculate the PAR, relative risk (RR) was obtained from systematic reviews, meta-analyses, and observational studies indexed in international journal databases (Cochrane Database of Systematic Reviews, Embase, and PubMed), published in English, between 1990 to 2017. The search query consisted of the following MeSH (Medical Subject Headings) terms: *“Renal Insufficiency*, *Chronic”*, *“Kidney Failure*, *Chronic”*, and *“Diabetes Mellitus”*. A handsearch of the reference lists of articles thus retrieved was also performed, and potentially eligible publications were added to the selection. Two independent investigators (GMRG and ENS) evaluated the quality of the selected articles, using the Assessment of Multiple Systematic Reviews (AMSTAR) tool.

The prevalence of diabetes was obtained from the National Health Survey [6]. This survey implemented a cluster sampling strategy and interviewed one adult resident (age >18 years) from each of 62,986 households distributed across Brazil. Detailed methodological information on the survey is available elsewhere [6].

A top-down approach was adopted for the identification, measurement, and valuation of the direct costs of CKD and ESKD, based on administrative data obtained from the SIA/SUS and SIH/SUS. These systems serve as a registry of all procedures reimbursed by the Brazilian Ministry of Health to health facilities (hospitals, clinics, and laboratories), public or private, that provide services to the Unified Health System. Direct costs were defined as those of outpatient (SIA/SUS) and inpatient (SIH/SUS) procedures, such as doctor’s appointments, laboratory tests, medications, hospital admissions, treatment of complications, renal replacement therapy, and renal transplantation. Non-medical direct costs (patient transport, caregiver payment), indirect costs (absenteeism, presenteeism, and early death), and intangible costs (loss of ability to work, loss of quality of life, among others) were disregarded.

For analysis, direct costs were stratified by variable (sex, race/skin color, and age) and combined with International Classification of Diseases (ICD-10) codes for CKD (N18, N18.8, N18.9) and ESKD (N18.0). The variables sex, race/skin color, and age were divided into the following categories: a) sex–female and male; b) age– 18 to 29 years, 30 to 59 years, 60 to 64 years, 65 to 74 years, and >75 years; c) race/skin color–Asian, White, Native Brazilian, Brown (*pardo*), Black, and not reported. Specifically for the race/skin color variable, data reallocation was required, as 24% of database entries belonged to the “not reported” category. Data reallocation was performed by calculating the percent share of the categories “Asian”, “White”, “Native Brazilian”, “Brown (*pardo*)”, “Black” in relation to total cost, disregarding costs for the “not reported” category; then, costs for the “not reported” category were redistributed based on the percent share calculated in the previous step.

Nominal values ​​for the 2010–2016 period were used, without any adjustment for inflation. This is common practice for Brazilian studies of public health costs which use administrative data, as the fee schedule of procedures offered by the Unified Health System is updated only irregularly and infrequently [19]. Costs were collected in Brazilian reais (R$) and subsequently converted to U.S. dollars (US$), at an exchange rate of US$1 = R$3.26, as of December 31, 2016, reported by the Central Bank of Brazil.

As this study used only aggregate information from government databases, which is in the public domain and offers no possibility of identifying individual subjects, approval by a review board of the National Research Ethics Committee (CEP/CONEP) was waived [20].

## Results

### Risk factors associated with chronic kidney disease

Table 1 lists the risk factors associated with chronic kidney disease and their relative risks (RR). Diabetes is the risk factor with the highest incidence in the CKD population (OR 1.75, 95% CI 1.62–1.89), and is associated with a six-fold increase in the risk of developing ESKD (RR 6.1, 95% CI 5.7–6.3). Among diabetics, women were at a higher risk of developing CKD and ESKD compared to men. Black race/skin color and age 65–75 years were also important factors that contributed to the development of ESKD.

**Table 1:** Relative risk (or odds ratio) of diabetes associated with chronic kidney disease (CKD) and end-stage kidney disease (ESKD) in adults (age >18 years), stratified by sex, race/skin color, and age.

		Chronic kidney disease			Ref.	End-stage kidney disease			Ref.
				95% confidence interval				95% confidence interval	
Diabetes		OR	1.75	(1.62–1.89)	[1]	RR	6.1	(5.70–6.30)	[55]
Sex	Female	RR	3.34	(2.27–4.93)	[28]	RR	4.68	(3.58–6.11)	[56]
	Male	RR	2.84	(1.73–4.68)	[28]	RR	2.79	(2.17–3.58)	[56]
Race/skin color	Black		NA	NA		RR	1.53	(1.26–1.85)	[44]
Age (years)	65–74		NA	NA		RR	1.393	(1.18–1.64)	[26]

Note: OR: Odds ratio; RR: Relative risk; NA: Not available. Source: [1] Ene-Iordache et al, 2016; [55] Gregg EW et al, 2014; [28] Y. S, R, 2016; [56]. Hippisley-Cox, 2010; [44] Lewis, 2010; [26] Lorenzo, 2010.

No statistically significant associations were found between diabetes, CKD and ESKD for the following variables: age 18–29 years, 30–59 years, 60–64 years, or >75 years; and race/skin color Asian, White, Native Brazilian, or Brown (*pardo*).

Table 2 reports the calculated PAR for diabetes associated with CKD and ESKD stratified by sex, race/skin color, and age.

**Table 2:** Population attributable risk (PAR) of diabetes attributable to chronic kidney disease (CKD) and end-stage kidney disease (ESKD) in adults (age >18 years), stratified by sex, race/skin color, and age.

		PAR, chronic kidney disease		PAR, end-stage kidney disease	
			95% confidence interval		95% confidence interval
Diabetes		0.043	0.035 to 0.051	0.234	0.219 to 0.241
Sex	Female	0.141	0.081 to 0.216	0.205	0.153 to 0.263
	Male	0.090	0.037 to 0.166	0.088	0.059 to 0.122
Race/skin color	Black	NA	NA	0.037	0.018 to 0.057
Age (years)	65–74	NA	NA	0.073	0.035 to 0.113

Note: PAR calculated by the author based on the relative risk measured by the following sources: [1] Ene-Iordache et al, 2016; [55] Gregg EW et al, 2014; [28] Y. S, R, 2016; [56]. Hippisley-Cox, 2010; [44] Lewis, 2010; [26] Lorenzo, 2010; and on the prevalence of diabetes reported by [6]. NA: Not available.

### Costs of chronic kidney disease and end-stage kidney disease

Table 3 presents the costs of CKD and ESKD, estimated at US$5.6 billion between 2010 and 2016, which corresponds to an annual cost of US$807 million. Group 3, which encompasses clinical procedures, accounted for 85% of cost of illness, mainly due to renal replacement therapy (hemodialysis and peritoneal dialysis). Chronic kidney disease is essentially treated on an outpatient basis. Accordingly, of all procedures, 91% were recorded in the Outpatient Information System (SIA/SUS), and only 9% in the Hospital Information System (SIH/SUS).

**Table 3:** Costs of chronic kidney disease (CKD) and end-stage kidney disease (ESKD) in the adult population of Brazil, stratified by information system, proportion of procedures done via the Unified Health System (SUS), and type of procedure. Brazil, 2010–2016.

Procedure group	Cost of illness recorded in SIA/SUS (U.S. dollars in thousands)	Cost of illness recorded in SIH/SUS (U.S. dollars in thousands)	Proportion performed in SUS (%)	Type of procedure reported in SIA/SUS and SIH/SUS
02 –Diagnostic procedures	65,241	2	1.2	Urea, potassium, and phosphorus measurement.
03 –Clinical procedures	4,627,986	175,983	85.0	Adult hemodialysis, hemodialysis for hepatitis B, C, and HIV, and peritoneal dialysis.
04 –Surgical procedures	50,192	2,401	0.9	Placement of catheter for dialysis, creation of arteriovenous fistula, and dressing.
05 –Transplantation	11,374	324,373	5.9	Tests for waitlist inclusion, kidney transplantation (deceased-donor), and post-transplant complications.
06 –Medications	42,909	-	0.8	Sevelamer, calcitriol, and epoetin alfa.
07 –Orthotics, prosthetics, and special materials	338,661	-	6.0	Dilator for catheter placement, metal guidewire for catheter placement, subclavian catheter.
08 –Ancillary health care activities	13,594	-	0.2	Patient transport, food allowance, and chaperone transport.
Total	5,149,958	502,759	100	-

Note: The table lists the top three procedures recorded with the highest frequency in the corresponding procedure group and information system. Source: Unified Health System Outpatient Information System (SIA/SUS) and Hospital Information System (SIH/SUS). Brazil, 2010–2016.

### Costs of chronic kidney disease and end-stage kidney disease attributable to diabetes

In Brazil, the cost of CKD and ESKD attributable to diabetes in the 2010–2016 period was US$1.2 billion, which amounts to US$180 million per year and 1.3% of all resources provided by the Ministry of Health for medium- and high-complexity care in 2016 (Table 4). Taking into consideration CKD and ESKD together, the cost attributable to diabetes accounts for 22% of the total cost of illness of CKD and ESKD in Brazil. When stratifying by disease severity, diabetes contributed 4.3% of the cost of CKD and 23.4% of the cost of ESKD (Table 5). The annual cost growth rates of CKD and ESKD over the period of analysis were 9.5% and 6.2% respectively.

**Table 4:** Cost of chronic kidney disease and end-stage kidney disease attributable to diabetes in the adult population (age >18 years) per year of analysis. Brazil, 2010–2016.

Year	Cost of CKD attributable to diabetes (U.S. dollars in thousands)		Cost of ESKD attributable to diabetes (U.S. dollars in thousands)	
		95% confidence interval		95% confidence interval
2010	1,415	1,179 to 1,666	136,677	128,340 to 140,744
2011	1,543	1,285 to 1,816	149,044	139,926 to 153,479
2012	1,882	1,567 to 2,215	168,008	157,730 to 173,007
2013	2,178	1,814 to 2,564	184,813	173,507 to 190,312
2014	2,427	2,021 to 2,857	195,303	183,355 to 201,114
2015	2,411	2,008 to 2,838	202,785	190,379 to 208,818
2016	2,672	2,225 to 3,145	208,771	195,998 to 214,983
Total	14,528	12,100 to 17,102	1,245,402	1,169,210 to 4,180,812
Annual mean	2,075	-	177,915	-

Note: Cost of illness calculation based on PAR and on values reported in the SUS information systems. CKD: Chronic kidney disease; ESKD: End-stage kidney disease. Source: Unified Health System Outpatient Information System (SIA/SUS) and Hospital Information System (SIH/SUS). Brazil, 2010–2016.

**Table 5:** Cost of chronic kidney disease and end-stage kidney disease attributable to diabetes in the adult population (age >18 years), stratified by sex, race/skin color, and age. Brazil, 2010–2016.

Variable	Categories	Cost of CKD attributable to diabetes		Cost of ESKD attributable to diabetes		Proportion of overall CKD costs attributable to diabetes (%)	Proportion of overall ESKD costs attributable to diabetes (%)
		(U.S. dollars in thousands)		(U.S. dollars in thousands)			
			95% confidence interval		95% confidence interval		
Diabetes		14,528	12,100 to 17,102	1,245,402	1,169,210 to 1,282,458	4.3	23.4
Sex	Female	19,460	11,288 to 29,831	456,158	340,664 to 586,715	5.8	8.6
	Male	17,966	7,539 to 32,954	272,212	56,297 to 377,659	5.3	5.1
Race/skin color	Black	NA	NA	25,620	13,201 to 41,429	NA	0.5
Age (years)	65–74	NA	NA	63,231	30,469 to 98,755	NA	1.2

Note: Cost of chronic kidney disease (CKD) and end-stage kidney disease (ESKD) based on PAR and on cost-of-illness data reported in the SUS information systems. NA: Not available.

Source: Unified Health System Outpatient Information System (SIA/SUS) and Hospital Information System (SIH/SUS). Brazil, 2010–2016.

### Cost of chronic kidney disease attributable to diabetes by sex, race/skin color, and age

In the 2010–2016 period, diabetic women accounted for US$475 million of the cost of CKD and ESKD, and diabetic men, for US$290 million. The costs attributed to Black race/skin color and age between 65 and 75 years amounted to US$25 million and US$63 million respectively in ESKD (Table 5).

## Discussion

This study shows that the costs of CKD and ESKD are increasing in Brazil, and accounted for approximately 3.5% of the Ministry of Health budget in 2016 (US$807 million per year), a share close to the expenditures of developed countries. In these countries, 2 to 3% of health expenditures are directed to ESKD, benefiting 0.1–0.2% of the overall population [9]. The costs of renal treatment represented 6.3% of the U.S. Medicare budget in 2010 [21], 4.1% of the health budget in Japan in 1996, 3.2% in South Korea in 2004 [22] and 1.3% of the UK National Health System (NHS) budget in 2011 [23].

In developed countries, the cost of treating ESKD is a major cost factor for health systems, with an annual growth rate of 6% to 12% in the last two decades, mainly due to hemodialysis and peritoneal dialysis [23]. In Brazil, the present study shows that the annual cost growth rates of CKD and ESKD over the period of analysis (2010–2016) were 9.5% and 6.2% respectively, and the number of patients on dialysis increased at a rate of 3.6% per year, especially among older adults [24]. These data suggest that health expenditures related to dialysis will continue to rise in the near future and highlighted the importance of preventive measures, especially those related to controlling the leading cause of CKD: diabetes [24].

In the present study, the relative risk (RR) of a diabetic developing CKD and ESKD was 1.75 (95%CI 1.62–1.89) and 6.10 (95%CI 5.7–6.3) respectively. This is consistent with previous research that has demonstrated a positive association between diabetes and chronic renal impairment [25,26,27,28]. In Brazil, 12% to 20% of the population on renal replacement therapy is estimated to have diabetes [24,29]; in developed countries, this figure may be as high as 40% [30].

Original findings of this study include the cost of CKD and ESKD attributable to diabetes, approximately US$180 million per year (22%). According to WHO, 30% of the cost of ESKD is attributable to diabetes [23]. Studies have shown that the monthly cost of managing CKD is US$1,200 and may exceed US$3,000 if diabetes and heart failure are also present [31].

Regarding the costs of the early stages of chronic renal impairment, classified in this study as CKD, a note of caution is warranted. These data may be underestimated in SUS information systems, mainly due to the lack of screening for CKD in patients at risk [1] and to delayed diagnosis [32]. A cohort study in Taiwan found that, although chronic kidney disease affected 11.9% of the adult population, only 3.5% of participants were aware of this diagnosis [33]. A cross-sectional study of 12 low- and middle-income countries showed that 31% of diabetics did not know they had CKD [1].

Another issue to be considered is the high mortality of patients with diabetes due to renal impairment and heart failure [31,33], which contributes to higher health-system expenditures. An analysis of diabetes mortality found that a substantial proportion of individuals who died of diabetes had renal failure, but the cause of death was coded as uncomplicated diabetes. This reveals a failure of the vital statistics information system in capturing chronic kidney disease [9].

Diabetes, with or without comorbid renal disease, has a clear and substantial impact on the health budget. A systematic review found that the annual direct cost of diabetes worldwide was US$825 billion, with China (US$170 billion), the United States (US$105 billion), India (US$73 billion), and Japan (US$37 billion) accounting for the highest diabetes-related expenditures [34]. In addition, it is estimated that 60% of the overall costs of diabetes are borne by developing and underdeveloped countries, which imposes costs on patients, their families, health systems, and national economies [34].

In the poorest parts of the world, the number of patients with CKD is believed to be growing rapidly, and a strong association between low levels of economic development and reduced availability of renal replacement therapy has been reported [9]. Especially in countries with weak economies, more than half of patients with ESKD receive no treatment whatsoever [35].

Within this context, authors have argued that, in low- and middle-income countries, screening programs to detect CKD in specific populations may reduce the burden and cost associated with this condition [35].

Income and poverty are directly related to social inequality in health [36]. Brazilian data on poverty and income distribution published by the Institute of Applied Economic Research (IPEA) in 2011 show that between 1995 and 2009, poverty rates declined in Brazil as a result of income distribution policies. However, sex and race inequalities mainly affect Brazilian black women [37].

In 2009, the income of Black women was 55% of their white male counterparts, demonstrating that this group remains at the bottom of Brazil’s social hierarchy [37]. National efforts to reduce poverty should be even greater, mainly when there are inequalities in terms of sex-race and spending on CKD.

### Sex

Our analysis by sex revealed that, in Brazil, with 51% of the population being women, the cost of CKD associated with diabetes was higher for women (US$475 million) than for men (US$290 million). This finding is explained by three factors: a) the higher prevalence of diabetes in women; c) the higher RR of CKD and ESKD in diabetic women; and c) physiological issues inherent to the female sex.

In Brazil, women have a higher proportion of reported diagnosis of diabetes than men (7.0% vs. 5.4% respectively) [6]. Also, a meta-analyses that analyses a global prevalence of CKD and ESKD by sex found a higher prevalence in females (12.1%) than in males (8.1%) [4]. In earlier reports, diabetes has generally been found to cause a greater adverse effect on major CKD risk factors in women than in men, requiring more consideration in clarifying the association between the sexes. Women with diabetes were more likely to have hypertension, dyslipidemia, and obesity, and were less likely to achieve the target value for glycated hemoglobin. In other words, women with diabetes had a higher chance of failing treatment targets than men [38].

In contrast a systematic review reported that the incidence of ESKD in diabetics is higher among white men (9 studies); however, in the black population, females are more likely to reach the end stage of renal disease (2 studies) [39]. The higher incidence of ESRD among white men may be explained partly by a higher prevalence of hypertension among males. Nevertheless, it has been shown that among older patients with diabetes who were starting hemodialises, mortality rates were higher among women than men (1.19 [95% CI 1.08–1.30]) [39].

Regarding mortality, women with diabetes are at higher risk of death compared to men [40]. In 1.577 patients (61% men, 60±15 years), men presented more CVD co-morbidity (OR: 1.88 [95% CI: 1.51–2.35]) but less diabetes mellitus (OR: 0.70 [0.55–0.89]) than women. Women with diabetes had a higher mortality risk (RR 2.93 [2.27–3.79]) than their male counterparts (RR 1.99 [1.52–2.59]), showing an interaction effect between sex and diabetes (RR 1.18 [0.37–2.00]) [40].

Some studies have reported possible physiological mechanisms that may explain the greater progression of CKD and ESKD in women with diabetes, such as a body composition characterized by lower lean mass; reduced estrogen levels in the postmenopausal period; changes in central adiposity; and insulin resistance [4,27,28,38].

In a recent literature review by Carrero *et al* (2018), the authors found that the epidemiology of CKD differs by sex, affecting women more than men, demonstrating that the effects of longer life expectancy on the natural decline in GFR, hormonal aspects, as well as the potential underdiagnosis of CKD, may be partly responsible for the higher prevalence of CKD in women. Furthermore, the authors suggest that the quality of life of women with CKD and ESKD is worse than that of men [41].

These discoveries provide insights that can be used to reduce access disparities for patients with CKD, which may be related in both sexes. In Brazil, access to health services seems not to be related to better or worse CKD care. Indicators of access to public health services show that women have more doctor appointments, examinations and take more SUS-funded basic medication than men [42]”.

### Race/Skin color

The cost of ESKD associated with diabetes in the Black population was estimated at US$25 million, and this population was 53% more likely to develop CKD than non-diabetics. Several findings converge to increase the RR of progression to ESKD in the Black population [43,44]. In Brazil, the Continuous National Household Sample Survey (2016) demonstrated that, between 2012 and 2016, the percentage of Whites in the population fell from 46.6% to 44.2%, while the percentage of Brown (*pardo*) individuals rose from 45.3% to 46.7% and Blacks from 7.4% to 8.2%.

Although our study looked for a risk association between diabetes and CKD in the White, Brown (*pardo*), Asian, and Native Brazilian subpopulations, no such combination was found. Most population-based international studies specify only white, nonwhite, or black ethnicity, which makes them difficult to correlate directly with the race/skin color classification used in the Brazilian National Health Survey (2013) and the Unified Health System information systems.

A Brazilian study that investigated the prevalence of self-reported medical diagnosis of CKD found that the highest prevalence among racial groups was presented by individuals who self-identified as Brown (*pardo*), a population not considered in the cost analysis [32]. Another Brazilian cohort found that CKD was predominantly prevalent in the black, Brown (*pardo*), and Native Brazilian populations [7].

Several causes are implicated in the higher prevalence of CKD and ESKD in the Black population with diabetes. Black patients are more likely to develop kidney disease because of the higher frequency of glomerulonephritis, hypertension, and diabetic nephropathy [45], as well as genetic predispositions and environmental factors such as smoking and diet [39]. In addition, social inequalities may predispose to increased exposure to CKD [7].

Studies have shown that individuals with low socioeconomic status are at 60% greater risk of developing CKD than those with a high socioeconomic level [2]. International findings show that Black British and Asians in the United Kingdom, Hispanics in the U.S., and indigenous populations in Australia, New Zealand, and Canada are at increased risk of developing CKD and ESKD because of disadvantages associated with racial background [46]. In Brazil, unemployed and illiterate persons are more likely to be black and Brown (*pardo*), with a consequent impact on the distribution of chronic noncommunicable diseases (NCDs) [11].

Brazilian studies demonstrate that racism is a determining factor in access to SUS health services, primarily for Black women that suffer from sex and race inequalities. The results reveal that white women account for 15.4% of access to health services, while Black women represent 7.9% [47]. In Brazil, the debate over prejudice and racial discrimination is incipient. Little is known about inter-relations between socioeconomic and racial inequalities, mainly among Black, Brown (*pardo*) and Native Brazilian individuals [11].

Although socioeconomic status plays a specific role in the incidence and prevalence of CKD, this does not fully account for the increased risk in the Black population. Cohort studies have shown that black patient have a 2.4% higher risk (95%CI 1.9–3.0) of ESKD when compared to whites, even after adjusting for age, sex, educational attainment, income, smoking, history of hypertension, diabetes, and other clinical conditions [44,48,49]. An association between obesity and ESKD in Black women was proposed by a longitudinal study of U.S. data. The findings suggest that Black women are at higher risk of ESKD than White women, and that a possible explanation would be the high prevalence of overweight and obesity in this group [43].

### Age

In our analysis of diabetes-related ESKD in the 65-to-75 age range, total Ministry of Health spending on outpatient and inpatient procedures amounted to US$63 million between 2010 and 2016. According to the methodology employed, this expenditure is influenced by the prevalence of diabetes in the Brazilian population and by the relative risk of developing ESKD in the elderly diabetic population. The population aged 65 years or older accounted for 7.38% of the total population, according to the 2010 Brazilian Census conducted by the Brazilian Institute of Geography and Statistics (IBGE). In the other age groups, no measures of association (RR or Odds Ratio) suggesting a positive correlation between diabetes and CKD or ESKD were found.

It is worth noting that, in Brazil, the age structure of the population is changing rapidly; the proportion of children and young people is declining, while both the proportion of older adults and their life expectancy are increasing [50]. The growing number of older adults in the population increases the burden of disease, especially NCDs. These transformations pose challenges for all sectors, and create a need to rethink the extent of service provision for the coming decades [50]. The increasing prevalence of diabetes as a function of age is a well-established phenomenon. Between ages 18 and 29, the prevalence of diabetes is 0.6%; for those aged 65 to 74, this rate can be as high as 19.9% [6], which predisposes to an increase in the burden of CKD.

In a study evaluating 66-year-olds, patients with both diabetes and CKD were found to have a greater decline in GFR (2.1 mL/min/1.73 m^2^ [95%CI 1.8–2.5] in women, 2.7 mL/min/1.73 m^2^ [95%CI 2.3–3.1] in men) when compared to non-diabetics with CKD [51]. Another study that evaluated predictors of CKD in diabetes concluded that, every 5 years, RR increases by 1.37 (P<0.001) [25].

It bears stressing that the cost findings of the present study may not reflect the true weight of ESKD in older adults with diabetes. Studies show that the burden of untreated renal impairment is significantly greater in the elderly than in younger people [52] and that many older adults appear not to benefit from renal replacement therapy, to the detriment of quality of life and high mortality as a result of cardiovascular complications [53].

Although the present analysis did not set out to calculate direct costs unrelated to health, indirect costs, and intangible costs associated with CKD, these costs do have a direct impact on individuals, their families, health systems, local governments, and global economies [23,54].

## Strengths and limitations

The use of information from the Outpatient Information System (SIA/SUS) and the Hospital Information System (SIH/SUS) provides a greater degree of comprehensiveness to this study, because these systems cover all health procedures paid for by the Ministry of Health—a federal agency that funds the majority of medium- and high-complexity care provided by the Unified Health System. The 7-year period of analysis, between 2010 and 2016, helped mitigate the possible confounding impacts of economic downturns and reduced investment in the health sector in specific years.

Limitations of the study include the fact that, when comparing the costs of CKD in different countries, methodological heterogeneity and different epidemiological characteristics must be taken into consideration. Furthermore, we were unable to calculate *per capita* costs, since Brazilian information systems count procedures rather than individuals. As we could not calculate relative risks for all of the desired variables of interest, the costs of CKD attributable to diabetes may have been underestimated.

## Conclusion

The costs of CKD and ESKD attributable to diabetes were estimated at US$1.2 billion in the 7-year period of analysis, which amounts to US$180 million per year on average. Diabetes currently accounts for 22% of annual expenditures on CKD and ESKD in the budget of the Brazilian public health system, with an upward trend. Diabetes accounted for a greater share of ESKD costs than CKD costs.

Female sex, age 65 to 75 years, and self-reported Black race/skin color, regardless of combinations thereof, contributed to an increase in the costs of CKD and ESKD (US$475 million, US$63 million, and US$25 million respectively). Explanations include the increasing prevalence of diabetes, genetic predispositions, social determinants, and changes in the age structure of the Brazilian population.

These findings may contribute to strengthening the promotion, prevention, surveillance and treatment of diabetes, given that it is a significant risk factor for CDK. It is also important to consider that in Brazil, women, Blacks and the elderly constitute a vulnerable population and may benefit from public health policies related to CKD and ESKD care.

In Brazil, public funding is essential in treating CKD, since middle-income earners cannot afford a renal replacement therapy. As such, for the Ministry of Health, which funds most CKD and ESKD treatment, implementing specific public policies to reduce the growth of the disease is an opportunity to mitigate the economic impacts, since CKD and ESKD-related expenses are growing at an annual rate of 6%.

There is a pressing need to discuss strategies for CKD screening in populations at higher risk, such as those identified in the study, in view of the serious implications of this condition for the budgetary sustainability of the Brazilian public health system.

Further research of this nature should be conducted in developing countries to monitor the progression of health spending and provide inputs for decision-making, helping improve the allocation of public resources.

## Supporting information

S1 Appendix Study data in XLSX format. (XLSX)

## References

[1] Ene-Iordache B, Perico N, Bikbov B, Carminati S, Remuzzi A, Perna A, et al Chronic kidney disease and cardiovascular risk in six regions of the world (ISN-KDDC): A cross-sectional study. Lancet Glob Heal [Internet]. Ene-Iordache et al. Open Access article distributed under the terms of CC BY-NC-ND; 2016;4(5):e307–19. Available from: 10.1016/S2214-109X(16)00071-1 27102194

[2] Webster AC, Nagler E V., Morton RL, Masson P. Chronic Kidney Disease. Lancet [Internet]. Elsevier Ltd; 2017;389(10075):1238–52. Available from: 10.1016/S0140-6736(16)32064-5 27887750

[3] Danaei G. Cardiovascular disease, chronic kidney disease, and diabetes mortality burden of cardiometabolic risk factors from 1980 to 2010: a comparative risk assessment. lancet Diabetes Endocrinol. England; 2014 8;2(8):634–47. 10.1016/S2213-8587(14)70102-0 24842598 PMC4572741

[4] Hill NR, Fatoba ST, Oke JL, Hirst JA, O’Callaghan CA, Lasserson DS, et al Global prevalence of chronic kidney disease—A systematic review and meta-analysis. Vol. 11, PLoS ONE. 2016. 10.1371/journal.pone.0158765 PMC4934905 27383068

[5] Levin A, Tonelli M, Bonventre J, Coresh J, Donner JA, Fogo AB, et al Global kidney health 2017 and beyond: a roadmap for closing gaps in care, research, and policy. Lancet. 2017;390(10105):1888–917. 10.1016/S0140-6736(17)30788-2 28434650

[6] Brasil. Instituto Brasileiro de Geografia e Estatística—IBGE. Pesquisa Nacional de Saúde [Internet]. Instituto Brasileiro de Geografia e Estatística—IBGE Rio de Janeiro; 2014 181 p. Available from: http://biblioteca.ibge.gov.br/visualizacao/livros/liv91110.pdf

[7] Barreto SM, Ladeira RM, Duncan BB, Schmidt MI, Lopes AA, Benseñor IM, et al Chronic kidney disease among adult participants of the ELSA-Brasil cohort: Association with race and socioeconomic position. J Epidemiol Community Health. 2015;70(4):380–9. 10.1136/jech-2015-205834 26511886

[8] Marinho AWGB, Penha A da P, Silva MT, Galvão TF. Prevalência de doença renal crônica em adultos no Brasil: revisão sistemática da literatura. Cad Saúde Coletiva [Internet]. 2017;25(3):379–88. Available from: http://www.scielo.br/scielo.php?script=sci_arttext&pid=S1414-462X2017000300379&lng=pt&tlng=pt

[9] Jha V, Garcia-Garcia G, Iseki K, Li Z, Naicker S, Plattner B, et al Chronic kidney disease: Global dimension and perspectives. Lancet [Internet]. Elsevier Ltd; 2013;382(9888):260–72. Available from: 10.1016/S0140-6736(13)60687-X 23727169

[10] Zhou B, Lu Y, Hajifathalian K, Bentham J, Di Cesare M, Danaei G, et al Worldwide trends in diabetes since 1980: A pooled analysis of 751 population-based studies with 4.4 million participants. Lancet [Internet]. NCD Risk Factor Collaboration. Open Access article distributed under the terms of CC BY; 2016;387(10027):1513–30. Available from: 10.1016/S0140-6736(16)00618-8 27061677 PMC5081106

[11] Schmidt MI, Duncan BB, E Silva GA, Menezes AM, Monteiro CA, Barreto SM, et al Chronic non-communicable diseases in Brazil: Burden and current challenges. Lancet. 2011;377(9781):1949–61. 10.1016/S0140-6736(11)60135-9 21561658

[12] Sesso RC, Lopes AA, Thomé FS, Lugon JR, Martins CT. Brazilian Chronic Dialysis Survey 2016. J Bras Nefrol [Internet]. 2017;39(3):261–6. Available from: 10.5935/0101-2800.20170049 29044335

[13] Cherchiglia ML, Gomes IC, Alvares J, Guerra Júnior A, Acúrcio F de A, Andrade EIG, et al Determinantes dos gastos com diálises no Sistema Único de Saúde, Brasil, 2000 a 2004. Cad Saude Publica [Internet]. 2010;26(8):1627–41. Available from: http://www.scielo.br/scielo.php?script=sci_arttext&pid=S0102-311X2010000800016&lng=pt&tlng=pt 21229221 10.1590/s0102-311x2010000800016

[14] de Oliveira ML, Santos LMP, da Silva EN. Bases metodológicas para estudos de custos da doença no Brasil. Rev Nutr. 2014;27(5):585–95.

[15] Paim J, Travassos C, Almeida C, Bahia L, MacInko J. The Brazilian health system: history, advances, and challenges. Lancet [Internet]. Elsevier Ltd; 2011;377(9779):1778–97. Available from: 10.1016/S0140-6736(11)60054-8 21561655

[16] De Oliveira ML, Santos LMP, Silvada EN. Direct healthcare cost of obesity in brazil: An application of the cost-of-illness method from the perspective of the public health system in 2011. PLoS One. 2015;10(4):1–15. 10.1371/journal.pone.0121160 PMC4382114 25830909

[17] International Monetary Fund. World economic outlook (International Monetary Fund) [Internet]. Washington, DC; 2017. p. 1–304. Available from: https://academic.oup.com/esr/article-lookup/doi/10.1093/esr/jcn046

[18] Silva Junior GB da, Bentes ACSN, Daher EDF, Matos SMA de. Obesity and kidney disease. J Bras Nefrol [Internet]. 2017;39(1):65–9. Available from: 10.5935/0101-2800.20170011 28355395

[19] Xavier DB, Ramalho WM, da Silva EN. Spending on Bariatric Surgery in the Unified Health System from 2010 to 2014: a Study Based on the Specialist Hospitals Authorized by the Ministry of Health. Obes Surg. Obesity Surgery; 2017;27(3):641–8. 10.1007/s11695-016-2327-5 27522602

[20] Ministério da Saúde. Resolução N^o^ 510, de 07 de Abril de 2016. Normas para Pesqui ciêntificas humanas e sociais [Internet]. 2016;58(12):1–10. Available from: http://conselho.saude.gov.br/resolucoes/2016/reso510.pdf%0Ahttp://conselho.saude.gov.br/resolucoes/2016/Reso510.pdf

[21] Collins AJ, Foley RN, Gilbertson DT, Chen S-C. United States Renal Data System public health surveillance of chronic kidney disease and end-stage renal disease. Kidney Int Suppl [Internet]. Nature Publishing Group; 2015;5(1):2–7. Available from: http://linkinghub.elsevier.com/retrieve/pii/S2157171615321006 10.1038/kisup.2015.2 PMC4455192 26097778

[22] Martins D, Tareen N, Zadshir A, Pan D, Vargas R, Nissenson A, et al The association of poverty with the prevalence of albuminuria: data from the Third National Health and Nutrition Examination Survey (NHANES III). Am J Kidney Dis [Internet]. 2006;47(6):965–71. Available from: http://www.pubmedcentral.nih.gov/articlerender.fcgi?artid=3863615&tool=pmcentrez&rendertype=abstract 10.1053/j.ajkd.2006.02.179 16731291 PMC3863615

[23] Couser WG, Remuzzi G, Mendis S, Tonelli M. The contribution of chronic kidney disease to the global burden of major noncommunicable diseases. Kidney Int [Internet]. Nature Publishing Group; 2011;80(12):1258–70. Available from: http://linkinghub.elsevier.com/retrieve/pii/S0085253815550047 10.1038/ki.2011.368 21993585

[24] de Moura L, Prestes IV, Duncan BB, Thome FS, Schmidt MI. Dialysis for end stage renal disease financed through the Brazilian National Health System, 2000 to 2012. BMC Nephrol. 2014;15:111 10.1186/1471-2369-15-111 25008169 PMC4099158

[25] De Cosmo S, Viazzi F, Pacilli A, Giorda C, Ceriello A, Gentile S, et al Predictors of chronic kidney disease in type 2 diabetes. Medicine (Baltimore) [Internet]. 2016;95(27):e4007 Available from: http://content.wkhealth.com/linkback/openurl?sid=WKPTLP:landingpage&an=00005792-201607050-00022 27399078 10.1097/MD.0000000000004007 PMC5058807

[26] Lorenzo V, Boronat M, Saavedra P, Rufino M, MacEira B, Novoa FJ, et al Disproportionately high incidence of diabetes-related end-stage renal disease in the Canary Islands. An analysis based on estimated population at risk. Nephrol Dial Transplant. 2010;25(7):2283–8. 10.1093/ndt/gfp761 20064954

[27] Yu MK, Katon W, Young BA. Associations between sex and incident chronic kidney disease in a prospective diabetic cohort. Nephrology. 2015;20(7):451–8. 10.1111/nep.12468 25807970 PMC4465880

[28] Tsai W-C, Wu H-Y, Peng Y-S, Ko M-J, Wu M-S, Hung K-Y, et al Risk Factors for Development and Progression of Chronic Kidney Disease. Medicine (Baltimore) [Internet]. 2016;95(11):e3013 Available from: http://content.wkhealth.com/linkback/openurl?sid=WKPTLP:landingpage&an=00005792-201603150-00021 26986114 10.1097/MD.0000000000003013 PMC4839895

[29] Cherchiglia ML, Gomes IC, Alvares J, Guerra Junior A, Acúrcio F de A, Andrade EIG, et al Determinantes dos gastos com diálises no Sistema Único de Saúde, Brasil, 2000 a 2004. Cad Saude Publica [Internet]. 2010;26(8):1627–41. Available from: http://www.scielo.br/scielo.php?script=sci_arttext&pid=S0102-311X2010000800016&lng=pt&nrm=iso&tlng=pt 21229221 10.1590/s0102-311x2010000800016

[30] Atkins RC, Zimmet P. Diabetic kidney disease: Act now or pay later? “World Kidney Day” March 11th 2010. Arch Cardiol Mex [Internet]. Elsevier Inc.; 2010;80(1):44–7. Available from: 10.1053/j.ajkd.2009.12.001 21147564

[31] Collins AJ, Foley RN, Herzog C, Chavers B, Gilbertson D, Ishani A, et al United States Renal Data System 2008 Annual Data Report Abstract. Am J Kidney Dis [Internet]. National Kidney Foundation, Inc.; 2009;53(1 SUPPL.):A6–7. Available from: 10.1053/j.ajkd.2008.10.005 19111206

[32] Moura L de, Andrade SSC de A, Malta DC, Pereira CA, Passos JEF. Prevalência de autorrelato de diagnóstico médico de doença renal crônica no Brasil: Pesquisa Nacional de Saúde, 2013. Rev Bras Epidemiol [Internet]. 2015;18(suppl 2):181–91. Available from: http://www.scielo.br/scielo.php?script=sci_arttext&pid=S1415-790X2015000600181&lng=pt&tlng=pt27008613

[33] Wen CP, Cheng TYD, Tsai MK, Chang YC, Chan HT, Tsai SP, et al All-cause mortality attributable to chronic kidney disease: a prospective cohort study based on 462 293 adults in Taiwan. Lancet [Internet]. 2008;371(9631):2173–82. Available from: http://www.ncbi.nlm.nih.gov/pubmed/18586172 10.1016/S0140-6736(08)60952-6 18586172

[34] Seuring T, Archangelidi O, Suhrcke M. The Economic Costs of Type 2 Diabetes: A Global Systematic Review. Pharmacoeconomics. 2015;33(8):811–31. 10.1007/s40273-015-0268-9 25787932 PMC4519633

[35] Jha V, Arici M, Collins AJ, Garcia-Garcia G, Hemmelgarn BR, Jafar TH, et al Understanding kidney care needs and implementation strategies in low- and middle-income countries: conclusions from a “Kidney Disease: Improving Global Outcomes” (KDIGO) Controversies Conference. Kidney Int. 2016;90(6):1164–74. 10.1016/j.kint.2016.09.009 27884311

[36] Barros MBDA. Desigualdade social em saúde: revisitando momentos e tendências nos 50 anos de publicação da RSP. Rev Saúde Pública [Internet]. 2017;51(17):01–8. Available from: http://www.scielo.br/pdf/rsp/v51/pt_0034-8910-rsp-S1518-87872017051000156.pdf

[37] Brasil, Ipea I de PEA. Retrato das desigualdades de gênero e raça. 4a ed. Brasília; 2011;39.

[38] Shen Y, et al Diabetes mellitus as a risk factor for incident chronic kidney disease and end-stage renal disease in women compared with men: a systematic review and meta-analysis. Endocrine [Internet]. S. Wang, Department of Endocrinology, Affiliated Zhongda Hospital of Southeast University, Nanjing, China; 2016 1 1 [cited 2017 Oct 18];55(1):66–76. Available from: http://link.springer.com/10.1007/s12020-016-1014-6 10.1007/s12020-016-1014-6 27477292

[39] Narres M, Claessen H, Droste S, Kvitkina T, Koch M, Kuss O, et al The incidence of end-stage renal disease in the diabetic (compared to the non-diabetic) population: A systematic review. PLoS One. 2016;11(1):1–28. 10.1371/journal.pone.0147329 PMC4727808 26812415

[40] Carrero JJ, de Mutsert R, Axelsson J, Dekkers OM, Jager KJ, Boeschoten EW, et al Sex differences in the impact of diabetes on mortality in chronic dialysis patients. Nephrol Dial Transplant. England; 2011 1;26(1):270–6. 10.1093/ndt/gfq386 20621930

[41] Carrero JJ, Hecking M, Chesnaye NC, Jager KJ. Sex and gender disparities in the epidemiology and outcomes of chronic kidney disease. Nat Rev Nephrol [Internet]. Nature Publishing Group; 2018;14(3):151–64. Available from: 10.1038/nrneph.2017.181 29355169

[42] Pinheiro RS, Viacava F, Travassos C, Brito A dos S. Gênero, morbidade, acesso e utilização de serviços de saúde no Brasil. Cien Saude Colet [Internet]. 2002;7(4):687–707. Available from: http://www.scielo.br/scielo.php?script=sci_arttext&pid=S1413-81232002000400007&lng=pt&tlng=pt

[43] Xue JL, Eggers PW, Agodoa LY, Foley RN, Collins AJ. Longitudinal Study of Racial and Ethnic Differences in Developing End-Stage Renal Disease among Aged Medicare Beneficiaries. J Am Soc Nephrol [Internet]. 2007;18(4):1299–306. Available from: http://www.jasn.org/cgi/doi/10.1681/ASN.2006050524 10.1681/ASN.2006050524 17329578

[44] Lewis EF, Claggett B, Parfrey PS, Burdmann EA, McMurray JJ V, Solomon SD, et al Race and ethnicity influences on cardiovascular and renal events in patients with diabetes mellitus. Am Heart J [Internet]. Elsevier Inc.; 2015;170(2):322–329.e4. Available from: 10.1016/j.ahj.2015.05.008 26299230

[45] Scialla JJ, Appel LJ, Astor BC, Miller ER, Beddhu S, Woodward M, et al Net endogenous acid production is associated with a faster decline in GFR in African Americans. Kidney Int [Internet]. Nature Publishing Group; 2012;82(1):106–12. Available from: 10.1038/ki.2012.82 22475819 PMC3540413

[46] Morton RL, Schlackow I, Mihaylova B, Staplin ND, Gray A, Cass A. The impact of social disadvantage in moderate-to-severe chronic kidney disease: An equity-focused systematic review. Nephrol Dial Transplant. 2016;31(1):46–56. 10.1093/ndt/gfu394 25564537

[47] Goes F, Rosendo E. MULHERES NEGRAS E BRANCAS E O ACESSO AOS SERVIÇOS PREVENTIVOS DE SAÚDE: uma análise sobre as desigualdades. 2011;571–9. Available from: https://repositorio.ufba.br/ri/bitstream/ri/12547/1/DISSER_PGENF_286_EMANUELLEFREITAS.pdf

[48] Lipworth L, Mumma MT, Cavanaugh KL, Edwards TL, Ikizler TA, E.Tarone R, et al Incidence and Predictors of End Stage Renal Disease among Low-Income Blacks and Whites. PLoS One. 2012;7(10):1–7. 10.1371/journal.pone.0048407 PMC3480508 23110237

[49] van den Beukel TO, de Goeij MCM, Dekker FW, Siegert CEH, Halbesma N. Differences in progression to esrd between black and white patients receiving predialysis care in a universal health care system. Clin J Am Soc Nephrol. 2013;8(9):1540–7. 10.2215/CJN.10761012 23846464 PMC3805080

[50] BRASIL M da S. Plano de ações estratégicas para o enfrentamento das doenças crônicas não transmissíveis (DCNT) no Brasil 2011–2022. Brasília; 2011. 160 p.

[51] Hemmelgarn BR, Zhang J, Manns BJ, Tonelli M, Larsen E, Ghali WA, et al Progression of kidney dysfunction in the community-dwelling elderly. Kidney Int. 2006;69(12):2155–61. 10.1038/sj.ki.5000270 16531986

[52] Hemmelgarn BR, James MT, Manns BJ, O’Hare AM, Muntner P, Ravani P, et al Rates of treated and untreated kidney failure in older vs younger adults. JAMA—J Am Med Assoc. 2012;307(23):2507–15. 10.1001/jama.2012.6455 22797451

[53] Ng BL, Anpalahan M. Management of chronic kidney disease in the elderly. Intern Med J. 2011;41(11):761–8. 10.1111/j.1445-5994.2011.02590.x 22077944

[54] Muka T.; et al The global impact of non-communicable diseases on healthcare spending and national income: a systematic review. Eur J Epidemiol [Internet]. O.H. Franco, Department of Epidemiology, Erasmus MC, University Medical Center Rotterdam, Rotterdam, Netherlands; 2015;30(4):251–77. Available from: http://www.embase.com/search/results?subaction=viewrecord&from=export&id=L603584954 10.1007/s10654-014-9984-2 25595318

[55] Gregg EW, Li Y, Wang J, Rios Burrows N, Ali MK, Rolka D, et al Changes in Diabetes-Related Complications in the United States, 1990–2010. N Engl J Med [Internet]. 2014;370(16):1514–23. Available from: http://www.nejm.org/doi/10.1056/NEJMoa1310799 10.1056/NEJMoa1310799 24738668

[56] Hippisley-Cox J, Coupland C. Predicting the risk of chronic kidney disease in men and women in England and Wales: prospective derivation and external validation of the QKidney® Scores. BMC Fam Pract [Internet]. 2010;11:13p–13p 1p. Available from: http://login.ezproxy.library.ualberta.ca/login?url = http://search.ebscohost.com/login.aspx?direct=true&db=rzh&AN=105073787&site=ehost-live&scope=site%5Cnhttp://download.springer.com/static/pdf/195/art%253A10.1186%252F1471-2296-11-49.pdf?originUrl=http:/%25 10.1186/1471-2296-11-49 PMC2905345 20565929

